# Efficacy and safety of immunotherapy rechallenge in second-line treatment after failure of first-line immune checkpoint inhibitors combined with chemotherapy in advanced gastric cancer: a retrospective study

**DOI:** 10.3389/fimmu.2025.1697712

**Published:** 2025-12-10

**Authors:** Fei Li, Wenjing Gong, Miaomiao Yang, Aina Liu

**Affiliations:** Department of Oncology, The Affiliated Yantai Yuhuangding Hospital of Qingdao University, Yantai, China

**Keywords:** advanced gastric cancer (AGC), second-line treatment, immunotherapy rechallenge, chemotherapy, PD-L1 expression

## Abstract

**Background:**

Gastric cancer is one of the most common malignant tumors worldwide, ranking fifth in incidence and fourth in mortality. While immune checkpoint inhibitor (ICI) combined chemotherapy has become the standard first-line treatment for advanced gastric cancer (AGC), most patients eventually experience disease progression. Currently, there is no unified consensus on second-line treatment strategies following failure of ICI combined chemotherapy. Immunotherapy rechallenge has shown potential efficacy in non-small cell lung cancer and melanoma, but its efficacy in AGC remains unclear. This retrospective study analyzed the effectiveness and safety of immunotherapy rechallenge versus chemotherapy in second-line treatment for AGC patients.

**Methods:**

We retrospectively analyzed 83 AGC patients who progressed after first-line treatment with ICI combined chemotherapy at Qingdao University Affiliated Yantai Yuhuangding Hospital between December 2021 and March 2025. Among them, 49 patients received immunotherapy rechallenge and 34 patients received chemotherapy as second-line therapy. Efficacy was assessed according to the RECIST v1.1 criteria, including Objective Response Rate (ORR), Disease Control Rate (DCR), Progression-Free Survival (PFS), Overall Survival (OS), and treatment-related adverse events (TRAEs) of grade 3 or higher.

**Results:**

Immunotherapy rechallenge demonstrated superior outcomes compared with chemotherapy, with higher ORR (30.6% vs. 6.0%) and DCR (83.7% vs. 38.3%). Median PFS and median OS were 4.57 vs. 2.20 months and 12.4 vs. 5.33 months, respectively. Cox regression analysis showed that second-line treatment modality, PD-L1 expression, and pathological type were independent prognostic factors for OS, whereas PFS was only influenced by treatment modality. In subgroup analysis, patients with CPS ≥1 derived significant benefit from immunotherapy rechallenge in both mPFS (4.83 vs. 2.20 months) and mOS (12.6 vs. 7.13 months, P<0.01), whereas no significant difference were observed in CPS<1 patients. Grade ≥3 TRAEs occurred in 26.5% of patients in the immunotherapy rechallenge group versus 35.3% in the chemotherapy group (p=0.049).

**Conclusion:**

For AGC patients who progressed after first-line ICI combined chemotherapy, immunotherapy rechallenge confers significant survival benefits compared to chemotherapy. Immunotherapy rechallenge is more effective in patients with high PD-L1 expression, suggesting its potential clinical application value as second-line treatment regimen.

## 1 Introduction

Gastric cancer is one of the most common cancers worldwide. Most patients are diagnosed at an advanced stage, resulting in poor overall prognosis with a 5-year survival rate of approximately 20% (1). China has a particularly high burden of gastric cancer, ranking first globally in both inciden and mortality rates, accounting for approximately 43.9% and 48.6%, respectively (2–4).

With the stagnation of chemotherapy development, limited options for targeted therapies, and unsatisfactory efficacy of immunotherapy alone, ICI combined with chemotherapy is increasingly being applied in AGC. Currently, first-line treatment for AGC is primarily based on HER2 status, MMR/MSI status, PD-L1 expression, and Claudin18.2 expression for patient stratification (5–8). However, AGC is characterized by rapid progression and high heterogeneity. Clinical trials such as CheckMate649 (9), ToGA (10), ATTRACTION-4 (11), and SPOTLIGHT/GLOW (12, 13) have reported a median PFS of approximately 6.7—10.4 months and a median OS of about 13.8—18 months. As a result, many patients eventually require second-line therapy.

Current second-line treatment strategies for AGC are guided by the pattern of disease progression following first-line therapy and by key molecular features, including HER2 status, PD-L1 expression, and MMR/MSI status (14, 15). Traditionally, for HER2-negative patients who have not received immunotherapy, Ramucirumab plus Paclitaxel remains the most commonly used regimen (16). For patients who progress after first-line immunotherapy, single-agent chemotherapy (such as Paclitaxel or Irinotecan) is also currently recommended (17).

As ICI combined with chemotherapy has become the new standard for first-line treatment of AGC (14, 18), an increasing number of patients experience disease progression after receiving such regimens. However, no consensus has been established regarding optimal second-line strategies for patients who fail first-line ICI combined with chemotherapy. Previous second-line regimens were primarily based on studies of single-agent chemotherapy or anti-angiogenic therapy combined with chemotherapy, and most clinical trials enrolled patients without prior exposure to immunotherapy. Therefore, there is a notable treatment gap in clinical practice, and evidence on effective therapies after failure of ICI plus chemotherapy remains limited. Therefore, identifying and evaluating second-line treatment modalities for this growing patient population has become an urgent clinical need.

To explore this issue, we conducted a retrospective study to evaluate the efficacy and safety of second-line treatment regimens for AGC patients who progressed after first-line ICI combined with chemotherapy. We collected data on 83 patients with AGC who received treatment at Qingdao University Affiliated Yantai Yuhuangding Hospital from December 2021 to March 2025. Through systematic analysis of these data, we aim to provide clinical evidence and references for second-line treatment in this population.

## 2 Methods

### 2.1 Study design

This single-center, retrospective study included 83 patients with AGC who experienced disease progression after first-line ICI combined with chemotherapy at Qingdao University Affiliated Yantai Yuhuangding Hospital from December 2021 to March 2025. Patients were divided into two groups based on their second-line treatment regimens: the immunotherapy rechallenge group and the chemotherapy group (Details of the first-line and second-line treatment regimens are shown in **Supplementary Table 1**). This study was approved by the Ethics Committee of Yuhuangding Hospital, Yantai [YYYIRB-IIT[2025]074]. All study procedures were strictly conducted under the standards and relevant regulations approved by the Ethics Committee.

### 2.2 Participants

Inclusion criteria: Age ≥18 years; histologically confirmed gastric cancer or adenocarcinoma of the gastroesophageal junction; disease progression after first-line ICI combined with chemotherapy, followed by at least two cycles of second-line treatment with evaluable efficacy; normal major organ function; complete clinical records and follow-up data available.

Exclusion Criteria: No ICI combined with chemotherapy received during first-line treatment; unclear tumor histological type or lack of pathological diagnostic evidence; missing imaging data, unable to assess efficacy; pregnant or lactating women; mental or neurological disorders affecting compliance; concomitant other malignant tumors; severe heart, liver, or kidney dysfunction; incomplete clinical data or loss to follow-up.

### 2.3 Data collection

Patient information was collected through the electronic medical record system, including demographic characteristics (age and gender), choice of second-line treatment regimen, duration of first-line treatment, ECOG PS, tumor histological type, site of metastasis, number of metastatic lesions, PD-L1 expression status, microsatellite instability status, history of gastrectomy, and serum markers (NLR/CEA/CA724).

### 2.4 Efficacy and safety assessments

Patients received heterogeneous second-line regimes reflecting real world clinical practice, including chemotherapy-only protocols using cytotoxic agents distinct from those in first-line therapy, as well as immunotherapy-rechallenge strategies in which either a different PD-1 inhibitor was combined with an alternative chemotherapy regimen or the same PD-1 inhibitor was continued with a modified chemotherapy combination (Details of first-line and second-line regimens are provided in **Supplementary Table 1**). Patients were followed at regular intervals throughout treatment. Radiological assessments are performed via CT or MRI after every two treatment cycles to evaluate treatment efficacy. Response was assessed according to the Response Evaluation Criteria in Solid Tumors (RECIST) version 1.1 and categorized as complete response (CR), partial response (PR), stable disease (SD), or progressive disease (PD), with the objective response rate (ORR) defined as the proportion of patients achieving CRor PR and the disease control rate (DCR) defined as the proportion of patients achieving CR, PR, or SD. Progression-free survival (PFS) was measured from the start of second-line treatment until disease progression or death from any cause, with patients without an event censored at their last follow-up. Overall survival (OS) was defined as the time from the start of second-line treatment to death from any cause, with censoring at the last follow-up for patients still alive. Treatment-related adverse events were monitored throughout the study and graded according to the Common Terminology Criteria for Adverse Events (CTCAE) version 5.0, with regular assessments at each treatment cycle, including laboratory tests, physical examinations, and symptom evaluations, and any events occurring between visits were promptly managed according to clinical judgment.

### 2.5 Statistical analysis

In this study, the two patient groups were well balanced with respect to baseline characteristics. Categorical variables were compared between treatment groups using chi-square or Fisher’s exact tests, as appropriate. Overall survival (OS) and progression-free survival (PFS) were estimated using the Kaplan–Meier method, and corresponding survival curves were plotted. Intergroup differences in survival were assessed with the log-rank test. Subgroup analyses were performed according to PD-L1 expression levels and the duration of first-line immune checkpoint inhibitor (ICI) therapy combined with chemotherapy to evaluate survival differences across subgroups. Hazard ratios (HRs) and 95% confidence intervals (CIs) were calculated using Cox proportional hazards regression models. Multivariate Cox models were further constructed to identify clinical factors independently associated with OS and PFS. All statistical analyses were conducted using R (version 4.3.1) and SPSS (version 26.0), and a two-sided p-value < 0.05 was considered statistically significant.

## 3 Results

### 3.1 Patient characteristics

**Figure 1:**
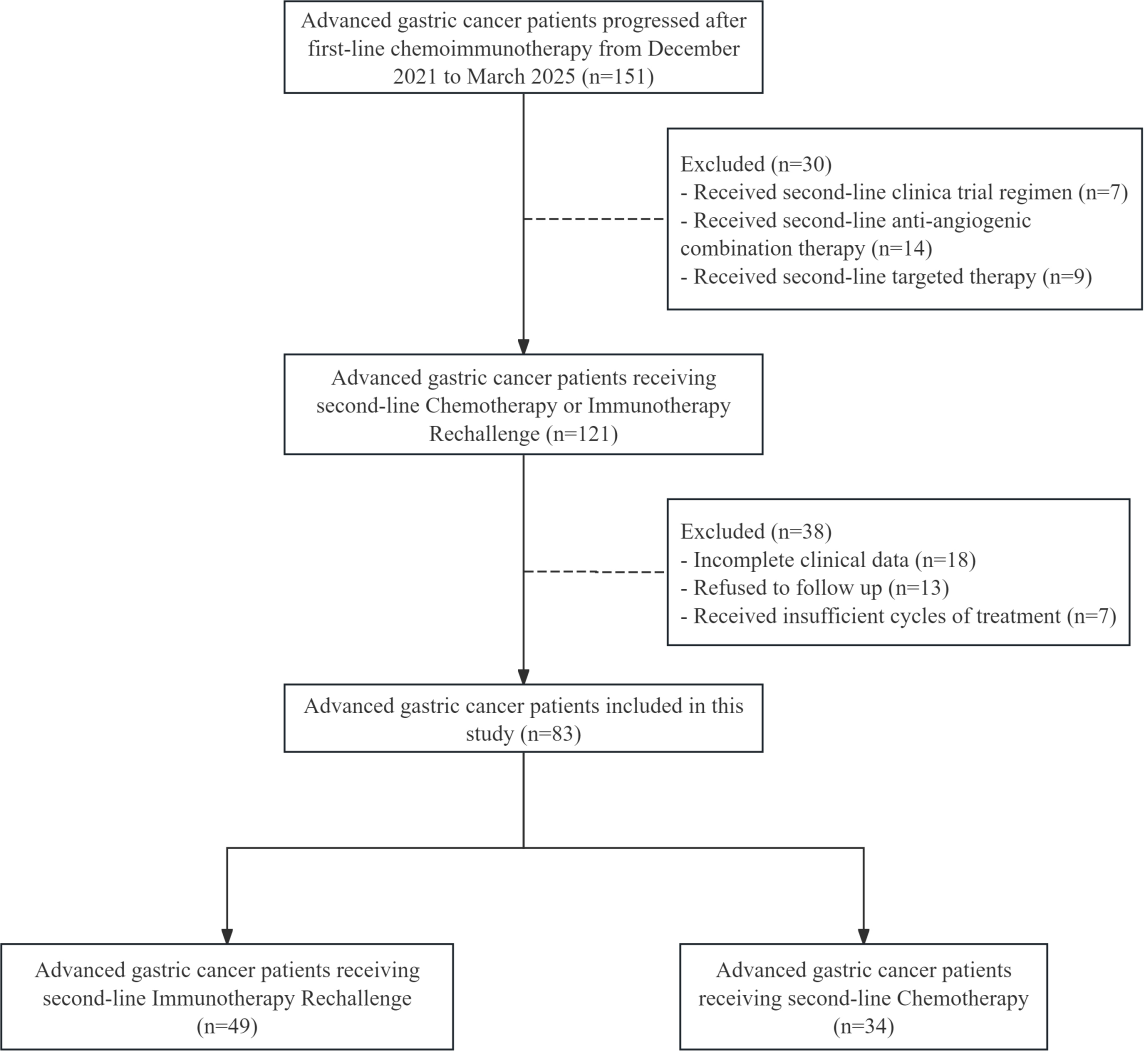
Flowchart of the recruitment process.

This retrospective study analyzed 83 patients with AGC who experienced disease progression after receiving first-line ICI combined with chemotherapy at Yuhuangding Hospital Affiliated to Qingdao University from December 2021 to March 2025. Among them, 49 patients received immunotherapy rechallenge as second-line treatment, while 34 patients received chemotherapy as second-line treatment (**Figure 1**). There were no significant differences in baseline characteristics between the two groups (**Table 1**).

**Table 1:** Baseline characteristics of the advanced gastric cancer patients.

Characteristics	Immunotherapy rechallenge (n=49)	Chemotherapy (n=34)	P value
Age, n (%)			**0.784**
<65	26 (31.3%)	17 (20.5%)	
≥65	23 (27.7%)	17 (20.5%)	
Gender, n (%)			**0.202**
Female	18 (21.7%)	8 (9.6%)	
Male	31 (37.3%)	26 (31.3%)	
ECOG performance status, n (%)			**0.885**
0-1	46 (55.4%)	33 (39.8%)	
2	3 (3.6%)	1 (1.2%)	
PD-L1 expression, n (%)			**0.960**
CPS <1	19 (22.9%)	13 (15.7%)	
CPS ≥1	30 (36.1%)	21 (25.3%)	
MSI, n (%)			**1.000**
MSS	48 (57.8%)	33 (39.8%)	
MSI-H	1 (1.2%)	1 (1.2%)	
Histology, n (%)			**0.607**
Adenocarcinoma	45 (54.2%)	33 (39.8%)	
Signet Ring Cell Carcinoma	4 (4.8%)	1 (1.2%)	
Number, n (%)			**0.470**
<2	15 (18.1%)	13 (15.7%)	
≥2	34 (41%)	21 (25.3%)	
History of gastrectomy, n (%)			**0.431**
Yes	12 (14.5%)	11 (13.3%)	
No	37 (44.6%)	23 (27.7%)	
Liver metastasis, n (%)			**0.846**
Yes	22 (26.5%)	16 (19.3%)	
No	27 (32.5%)	18 (21.7%)	
Peritoneum metastasis, n (%)			**0.457**
Yes	12 (14.5%)	6 (7.2%)	
No	37 (44.6%)	28 (33.7%)	
NLR, n (%)			**0.813**
<3	20 (24.1%)	13 (15.7%)	
≥3	29 (34.9%)	21 (25.3%)	
CEA, n (%)			**0.131**
<5	25 (30.1%)	23 (27.7%)	
≥5	24 (28.9%)	11 (13.3%)	
CA724, n (%)			**0.546**
<6.9	22 (26.5%)	13 (15.7%)	
≥6.9	27 (32.5%)	21 (25.3%)	

Bold values indicate the p-value.

### 3.2 Efficacy

**Figure 2:**
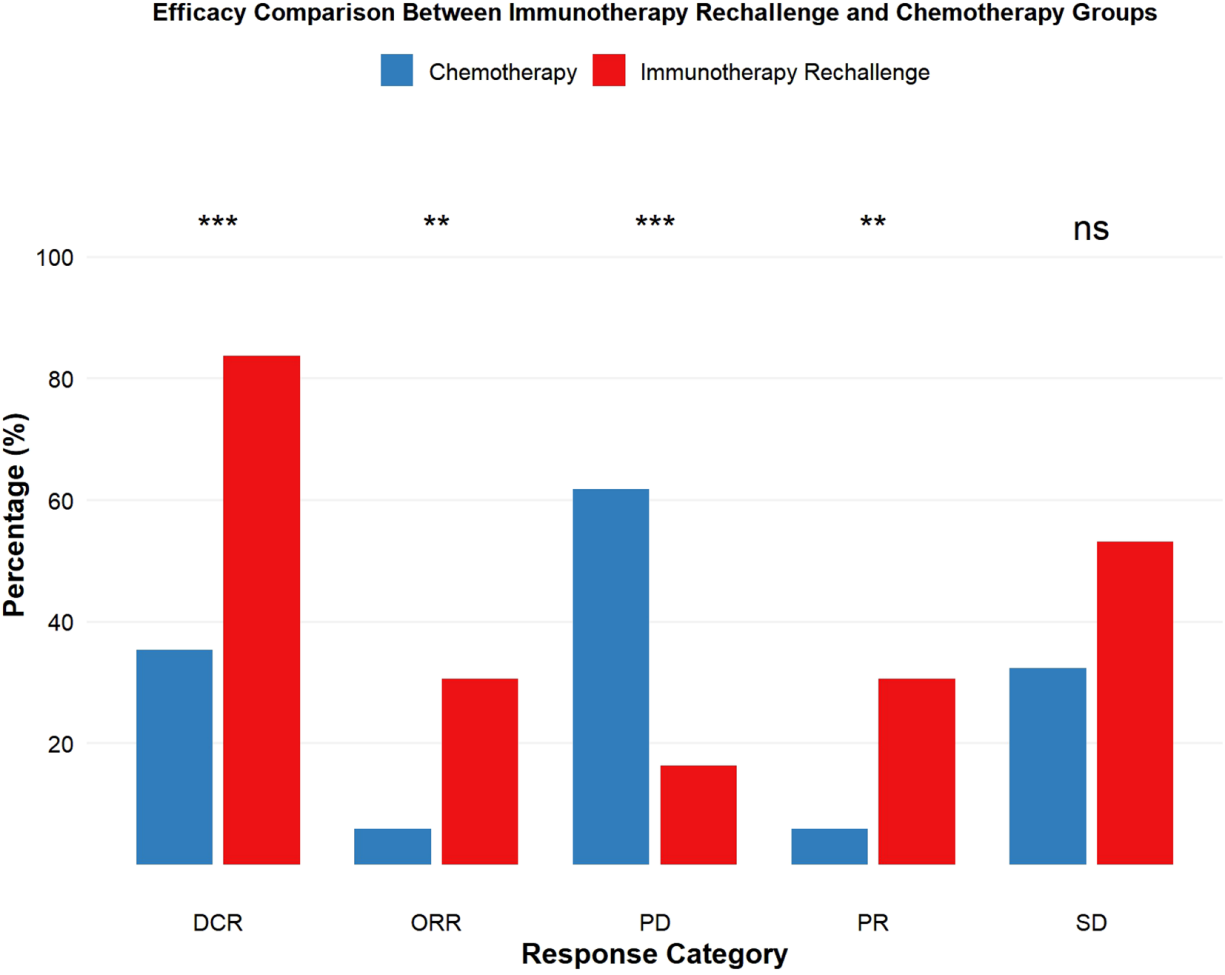
Efficacy comparison between immunotherapy rechallenge group and chemotherapy groups. ** corresponds to p < 0.01, and *** corresponds to p < 0.001.

**Figure 3:**
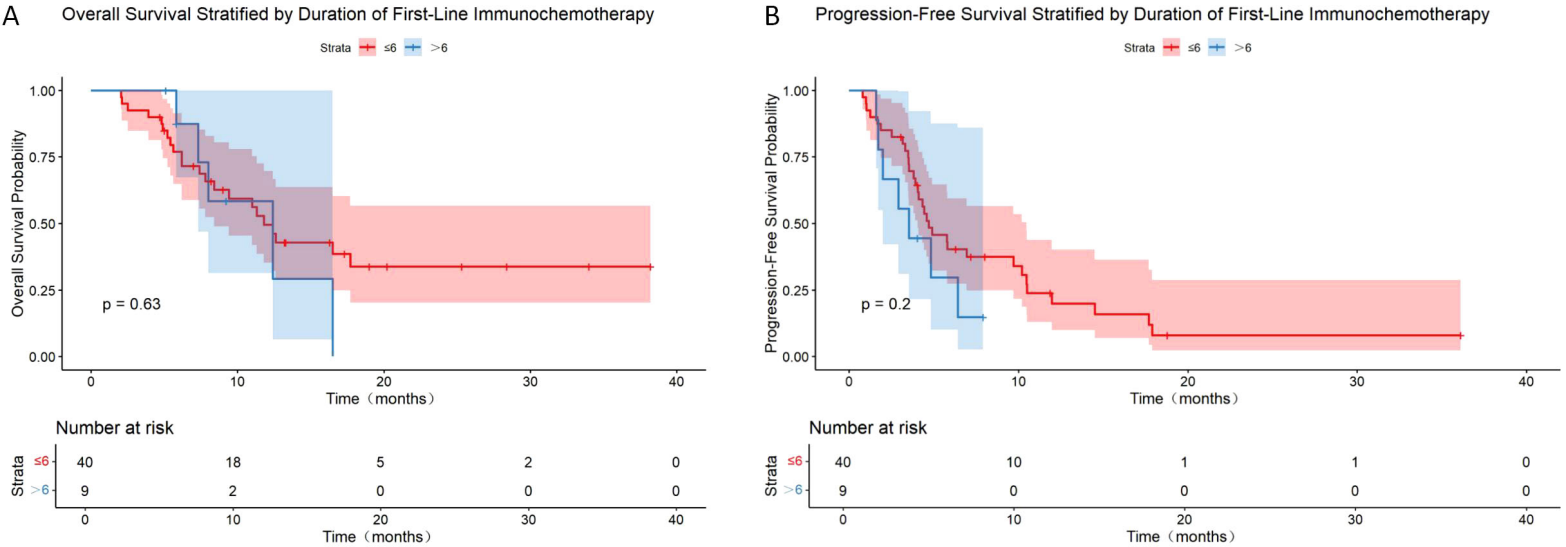
Kaplan–Meier curves of overall survival **(A)** and progression-free survival **(B)** by treatment group.

The efficacy results are summarized in **Table 2**; **Figure 2**. The objective response rate (ORR) was significantly higher in the immunotherapy rechallenge group than in the chemotherapy group (30.6% vs. 6.0%, p < 0.05), and the disease control rate (DCR) was also higher (83.7% vs. 38.3%, p < 0.001). Median overall survival (mOS) was 12.4 months (95% CI: 8.4–NA) in the immunotherapy rechallenge group, significantly longer than 5.33 months (95% CI: 3.53–10.10) in the chemotherapy group. Median progression-free survival (mPFS) showed a similar trend, with 4.57 months (95% CI: 3.91–9.70) versus 2.20 months (95% CI: 1.87–3.60) in the chemotherapy group (**Table 3**, **Figure 3**).

**Table 2:** Treatment response based on RECIST 1.1.

Efficacy	Immunotherapy rechallenge (n=49)	Chemotherapy (n=34)	P-value
CR, n (%)	0 (0%)	0 (0%)	–
PR, n (%)	15 (30.6%)	2 (6.0%)	0.006
SD, n (%)	26 (53.1%)	11 (32.3%)	–
PD, n (%)	8 (0.2%)	21 (61.7%)	<0.001
ORR (CR+PR)	15 (30.6%)	2 (6.0%)	0.006
DCR (CR+PR+SD), n (%)	41 (83.7%)	12 (38.3%)	<0.001

**Table 3:** Median PFS and median OS in immunotherapy rechallenge and chemotherapy groups.

	Immunotherapy rechallenge (months)	Chemotherapy (months)
Median OS in months (95%CI)	12.40 (8.4-NA)	5.33 (3.53-10.10)
Median PFS in months (95%CI)	4.57 (3.91-9.70)	2.20 (1.87-3.60)

To explore potential factors influencing OS and PFS, univariate and multivariate survival analyses were performed using Cox proportional hazards models in 83 patients (**Tables 4**, **5**). In univariate analysis of OS, second-line treatment (chemotherapy vs. immunotherapy rechallenge), PD-L1 expression (CPS < 1 vs. CPS ≥ 1), and pathological type (adenocarcinoma vs. signet ring cell carcinoma) were significantly associated with OS (p < 0.05), while other variables were not (p > 0.05). Variables with p < 0.1 were included in the multivariate model, which confirmed that second-line treatment, PD-L1 expression, and pathological type were independent prognostic factors for OS (p < 0.05). Notably, the signet ring cell carcinoma subgroup included only five patients, resulting in wide confidence intervals and potential discrepancies between univariate and multivariate analyses, suggesting possible bias or model instability. For PFS, univariate analysis identified only second-line treatment as a significant factor, which remained the sole independent prognostic factor in multivariate analysis.

**Table 4:** Univariate and multivariate analysis of clinical characteristics associated with overall survival (OS) in advanced gastric cancer patients.

Characteristics	Total (N)	Univariate analysis		Multivariate analysis	
		Hazard ratio (95% CI)	P value	Hazard ratio (95% CI)	P value
Treatment	83				
Immunotherapy Rechallenge	49	Reference		Reference	
Chemotherapy	34	2.376 (1.372 - 4.116)	**0.002**	2.088 (1.194 - 3.648)	**0.010**
Age	83				
<65	43	Reference			
≥65	40	0.676 (0.386 - 1.183)	0.170		
Gender	83				
Female	26	Reference			
Male	57	1.608 (0.855 - 3.022)	0.140		
ECOG performance status	83				
0-1	79	Reference		Reference	
2	4	0.000 (0.000 - Inf)	0.997	0.000 (0.000 - Inf)	0.996
PD-L1 expression	83				
CPS<1	32	Reference		Reference	
CPS≥1	51	0.543 (0.305 - 0.969)	**0.039**	0.464 (0.256 - 0.841)	**0.011**
MSI	83				
MSS	81	Reference			
MSIH	2	2.724 (0.367 - 20.199)	0.327		
Histology	83				
Adenocarcinoma	78	Reference		Reference	
Signet Ring Cell Carcinoma	5	0.320 (0.077 - 1.329)	0.117	0.212 (0.048 - 0.923)	**0.039**
Number	83				
<2	28	Reference			
≥2	55	1.289 (0.714 - 2.327)	0.399		
Historyofgastrectomy	83				
No	60	Reference		Reference	
Yes	23	1.670 (0.942 - 2.960)	0.079	1.303 (0.720 - 2.359)	0.383
Liver metastasis	83				
No	45	Reference			
Yes	38	1.064 (0.616 - 1.838)	0.823		
Peritoneum metastasis	83				
No	65	Reference			
Yes	18	0.731 (0.374 - 1.430)	0.360		
NLR	83				
<3	33	Reference			
≥3	50	1.207 (0.685 - 2.128)	0.515		
CEA	83				
<5	35	Reference		Reference	
≥5	48	1.613 (0.910 - 2.922)	0.100	1.505 (0.823 - 2.753)	0.184
CA724	83				
<6.9	35	Reference			
≥6.9	48	1.587 (0.905 - 2.784)	0.107		

Bold values indicate statistically significant differences.

**Table 5:** Univariate and multivariate analysis of clinical characteristics associated with progression-free survival (PFS) in advanced gastric cancer patients.

Characteristics	Total (N)	Univariate analysis		Multivariate analysis	
		Hazard ratio (95% CI)	P value	Hazard ratio (95% CI)	P value
Treatment	83				
Immunotherapy Rechallenge	49	Reference		Reference	
Chemotherapy	34	2.330 (1.409 - 3.852)	**< 0.001**	2.053 (1.239 - 3.400)	**0.005**
Age	83				
<65	43	Reference			
≥65	40	0.839 (0.519 - 1.357)	0.474		
Gender	83				
Female	26	Reference		Reference	
Male	57	1.631 (0.954 - 2.790)	0.074	1.321 (0.769 - 2.269)	0.314
ECOG performance status	83				
0-1	79	Reference		Reference	
2	4	0.000 (0.000 - Inf)	0.996	0.000 (0.000 - Inf)	0.996
PD-L1 expression	83				
CPS<1	32	Reference			
CPS≥1	51	0.680 (0.408 - 1.133)	0.138		
MSI	83				
MSS	81	Reference			
MSIH	2	2.836 (0.378 - 21.265)	0.311		
Histology	83				
Adenocarcinoma	78	Reference			
Signet Ring Cell Carcinoma	5	0.782 (0.313 - 1.956)	0.599		
Number	83				
<2	28	Reference			
≥2	55	1.024 (0.616 - 1.703)	0.926		
History of gastrectomy	83				
No	60	Reference			
Yes	23	1.152 (0.669 - 1.982)	0.610		
Liver metastasis	83				
No	45	Reference			
Yes	38	1.335 (0.829 - 2.150)	0.235		
Peritoneum metastasis	83				
No	65	Reference			
Yes	18	0.640 (0.357 - 1.150)	0.136		
NLR	83				
<3	33	Reference			
≥3	50	1.110 (0.678 - 1.819)	0.678		
CEA	83				
≥5	48	Reference			
<5	35	0.685 (0.420 - 1.116)	0.128		
CA724	83				
<6.9	35	Reference			
≥6.9	48	1.101 (0.680 - 1.783)	0.696		

Bold values indicate statistically significant differences.

### 3.3 Subgroup analysis

**Figure 4:**
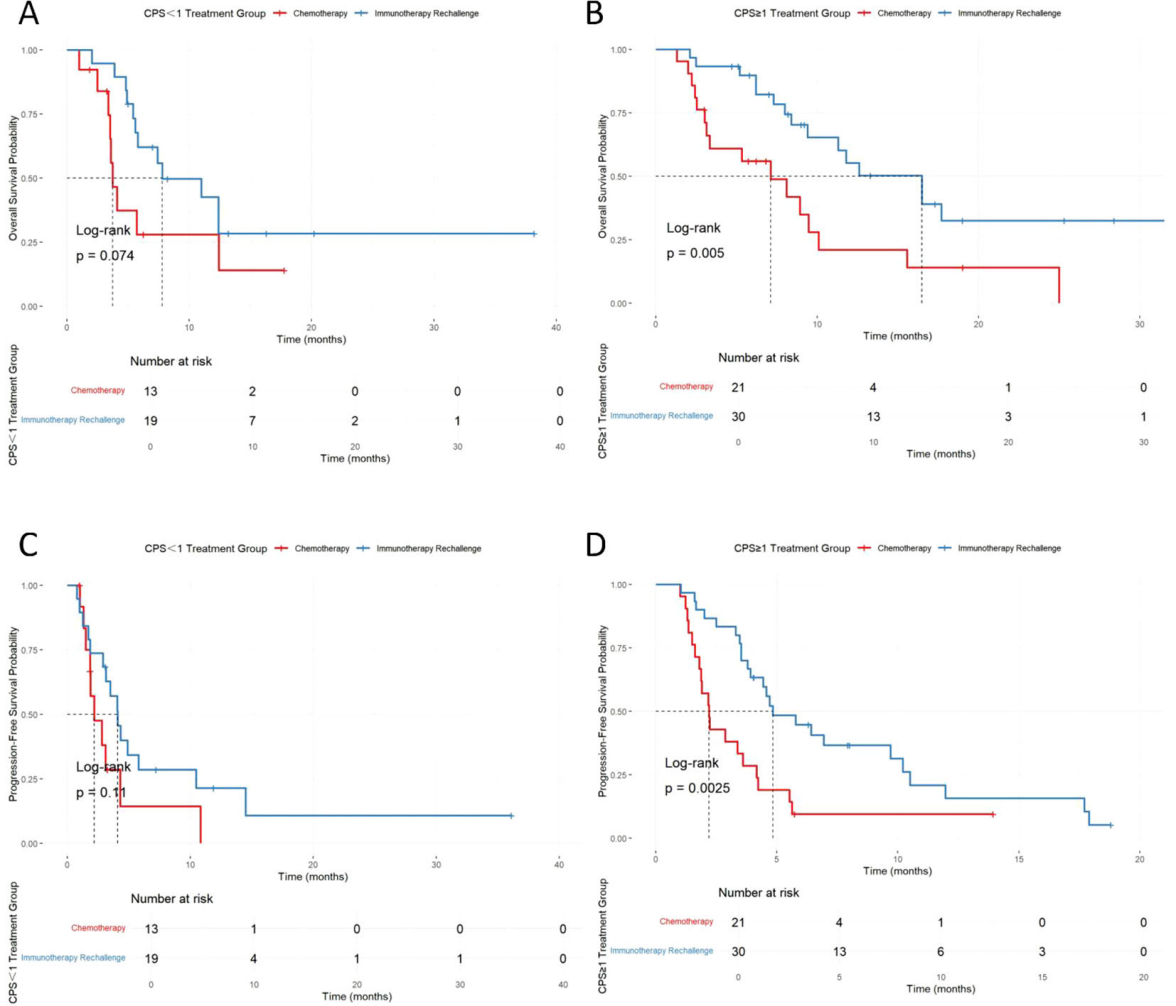
Kaplan–Meier curves of OS and PFS stratified by PD-L1 CPS status. **(A)** OS in patients with PD-L1 CPS <1. **(B)** OS in patients with PD-L1 CPS ≥1. **(C)** PFS in patients with PD-L1 CPS <1. **(D)** PFS in patients with PD-L1 CPS ≥1.

In patients with high PD-L1 expression (CPS ≥ 1), median OS (mOS) was 12.6 months (95% CI: 9.40–NA) in the immunotherapy rechallenge group, compared with 7.13 months (95% CI: 3.13–15.7) in the chemotherapy group. Median PFS (mPFS) was 4.83 months (95% CI: 3.91–10.5) versus 2.20 months (95% CI: 1.80–4.23), with statistically significant differences between groups (p = 0.0086 for OS; p = 0.0025 for PFS). In patients with low PD-L1 expression (CPS < 1), mOS was 7.8 months (95% CI: 5.60–NA) in the immunotherapy rechallenge group and 3.73 months (95% CI: 3.53–NA) in the chemotherapy group. Median PFS was 4.1 months (95% CI: 3.15–NA) versus 2.2 months (95% CI: 1.87–NA), but the differences were not statistically significant (p = 0.074 for OS; p = 0.11 for PFS) (**Table 6**, **Figure 4**).

**Table 6:** Median Overall Survival (OS) and Progression-Free Survival (PFS) stratified by PD-L1 CPS status and treatment regimen.

	PD-L1 CPS<1		PD-L1 CPS≥1	
	Immunotherapy rechallenge	Chemotherapy	Immunotherapy rechallenge	Chemotherapy
Median OS in months (95%CI)	7.8 (5.60-NA)	3.73 (3.53-NA)	12.60 (9.40-NA)	7.13 (3.13-15.7)
Median PFS in months (95%CI)	4.1 (3.15-NA)	2.2 (1.87-NA)	4.83 (3.91-10.5)	2.20 (1.80-4.23)

**Figure 5:**
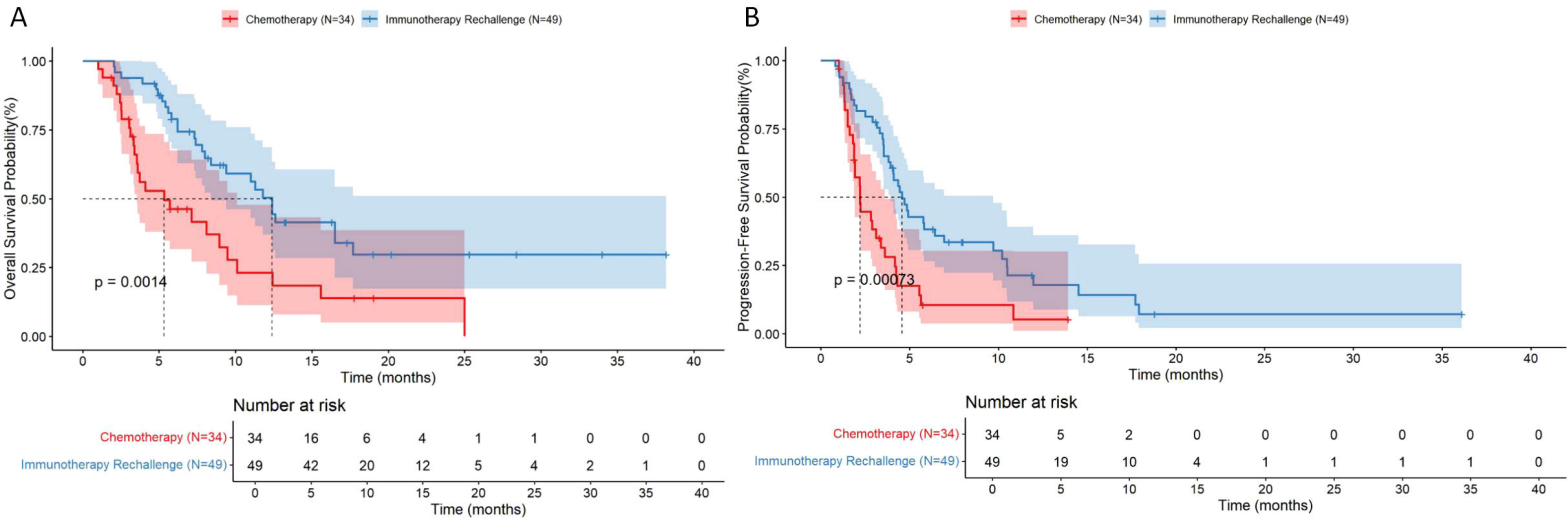
Kaplan–Meier curves of OS **(A)** and PFS **(B)** in immunotherapy rechallenge group stratified by duration of first-line immunochemotherapy.

To investigate the impact of first-line ICI combined with chemotherapy duration on the efficacy of second-line immunotherapy rechallenge, patients in the rechallenge group were divided into short-course (≤6 cycles) and long-course (>6 cycles) subgroups. Median PFS was 3.53 months (95% CI: 2–NA) and mOS was 12.4 months (95% CI: 7.3–NA) in the long-course group, compared with 4.7 months (95% CI: 4.05–10.47) and 11.8 months (95% CI: 8.4–NA) in the short-course group. No statistically significant differences were observed between the two subgroups for either mPFS or mOS (p = 0.20 for PFS; p = 0.63 for OS) (**Table 7**, **Figure 5**).

**Table 7:** Median Overall Survival (OS) and Progression-Free Survival (PFS) Stratified by Duration of First-Line Immunochemotherapy.

	Duration of first-line immunochemotherapy	
	≤ 6 cycles	> 6 cycles
Median OS in months (95%CI)	11.8 (8.4-NA)	12.4 (7.3-NA)
Median PFS in months (95%CI)	4.7 (4.046-10.47)	3.53 (2-NA)

### 3.4 Safety

There was no statistically significant difference in the overall incidence of treatment-related adverse events (TRAEs) between the immunotherapy rechallenge group (38/49, 77.6%) and the chemotherapy group (31/34, 91.2%) (p = 0.109), indicating a comparable overall risk of adverse events. The incidence of grade ≥3 severe adverse events was low in both groups—13 (26.5%) in the immunotherapy rechallenge group and 12 (35.3%) in the chemotherapy group—with no significant difference observed (p = 0.409), suggesting a similar risk of severe toxicity between the two regimens (**Table 8**). These findings indicate that immunotherapy rechallenge is generally well tolerated and does not substantially increase the risk of severe adverse events.

**Table 8:** Comparison of treatment-Related adverse events between groups.

Adverse events	Grade 1 or 2, n (%)		P-value	Grade 3 or 4, n (%)		P-value
	Immunotherapy rechallenge	Chemotherapy		Immunotherapy rechallenge	Chemotherapy	
Myelosuppression	28 (57.1%)	22 (64.7%)	0.505	9 (18.4%)	7 (20.6%)	0.813
Abnormal liver function	18 (36.7%)	19 (55.9%)	0.116	3 (6.1%)	4 (11.8%)	0.673
Abnormal renal function	1 (2.0%)	1 (2.9%)	1.000	0 (0%)	0 (0%)	NA
Decreased appetite	20 (40.8%)	15 (44.2%)	0.823	0 (0%)	0 (0%)	NA
Nausea and vomiting	7 (14.3%)	5 (14.7%)	1.000	2 (4.1%)	2 (5.9%)	1.000
Diarrhea	2 (4.1%)	2 (5.9%)	1.000	0 (0%)	1 (2.9%)	0.402
Fatigue	24 (49.0%)	17 (50.0%)	1.000	0 (0%)	0 (0%)	NA
Constipation	1 (2.0%)	2 (5.9%)	0.565	0 (0%)	0 (0%)	NA
Neurotoxicity	1 (2.0%)	0 (0%)	1.000	0 (0%)	0 (0%)	NA
Hypothyroidism	1 (2.0%)	0 (0.0%)	1.000	0 (0%)	0 (0%)	NA
Hypoparathyroidism	0 (0.0%)	1 (2.9%)	0.410	0 (0%)	0 (0%)	NA
Rash	2 (4.1%)	1 (2.9%)	1.000	0 (0%0	0 (0%)	NA
Adrenal insufficiency	0 (0.0%)	1 (2.9%)	0.410	0 (0%)	0 (0%)	NA

## 4 Discussion

In recent years, immune checkpoint inhibitors (ICI) combined with chemotherapy has become the standard first-line treatment for advanced gastric cancer (AGC). However, most patients still experience disease progression after treatment, and there is no unified consensus on second-line treatment strategies following failure of first-line ICI combined with chemotherapy. Several real-world studies have demonstrated that immunotherapy rechallenge can yield significant survival benefits in non-small cell lung cancer (19) and melanoma (20).

In this retrospective study of 83 AGC patients, immunotherapy rechallenge demonstrated superior efficacy compared with chemotherapy, with higher ORR (30.6% VS. 6.0%), DCR (83.7% VS. 38.3%), mPFS (4.57 VS. 2.20 months), and mOS (12.4 VS. 5.33 months), supporting its potential as a clinically superior second-line option. Notably, the incidence of ≥3-grade treatment-related adverse events was manageable (26.5% VS. 35.3%), challenging the traditional assumption that rechallenge phases are associated with more severe toxicity (21). Selection bias may contribute, as patients tolerating rechallenge often have favorable baseline immune status and prior tolerance to initial immunotherapy (20, 22). In addition, initial immunotherapy may induce persistent immune memory, allowing rapid and effective activation of antitumor immune responses upon re-exposure while potentially reducing immune-related toxicity (23, 24). Immune fatigue or tolerance after the initial treatment peak may also lead to reduced toxicity reactions during the immunotherapy rechallenge phase (25, 26).

In our study, PD-L1 expression levels correlated with treatment efficacy, with CPS ≥1,patients deriving the greatest benefit. Mechanically, high PD-L1 expression typically reflects an inflamed tumor microenvironment, such as CD8+ T cell infiltration and activation of the IFN-γ signaling pathway (27–29), which may facilitate rapid reactivation of immune memory during re-challenge (30). However, the exact immunologic mechanisms remain to be elucidated. PD-L1 alone has limitations due to its heterogeneous and dynamic expression, and single-time-point detection may not accurately reflect its true expression levels during treatment process or at the time of immunotherapy rechallenge (31, 32). Some PD-L1-negative patients also benefit from immunotherapy rechallenge, suggesting that combining PD-L1 with other immune-related biomarkers—such as tumor infiltrating lymphocytes, tumor mutational burdon, or IFN-γ signaling—may improve patient selection.

Although the number of treatment cycles did not show a statistically significant impact on PFS or OS, this observation underscores the need to investigate the relationship between immune kinetics and clinical efficacy. Certain potentially relevant variables, such as the interval between first-line ICI discontinuation and rechallenge, were not systematically collected, limiting further exploratory analysis.

When compared with historical second-line treatment studies, immunotherapy rechallenge demonstrated comparable or slightly improved survival outcomes, particularly in PD-L1 CPS ≥1 populations (33). These observations are broadly consistent with emerging data from solid tumors (34, 35), including gastrointestinal malignancies, supporting the feasibility and potential benefit of ICI rechallenge or sequential strategies. Most evidence comes from retrospective analyses and early-phase studies in other solid tumors, which suggest that selected patient populations may derive benefit, although prospective validation in gastric cancer is still needed.

Future prospective, multicenter, randomized trials are warranted to validate the efficacy and safety of ICI rechallenge. Integration of translational studies to explore immunologic mechanisms and predictive biomarkers will be essential. Our data suggest that patients with PD-L1 CPS ≥1 and favorable clinical status may represent an enriched population for rechallenge. Identification of additional biomarkers and careful patient selection will help optimize individualized treatment strategies.

In summary, immunotherapy rechallenge following failure of first-line ICI combined with chemotherapy demonstrates promising efficacy and manageable safety in AGC patients. Future integration of basic and clinical research to explore mechanisms, biomarkers, and patient selection criteria will help improve treatment outcomes and quality of life for these patients.

## Data Availability

The dataset contains confidential patient information and is not publicly available. Requests to access these datasets should be directed to nana4312@sina.com.
