# Supplementary material for: Efficacy and safety of immunotherapy rechallenge in second-line treatment after failure of first-line immune checkpoint inhibitors combined with chemotherapy in advanced gastric cancer: a retrospective study
Source: Front Immunol. 2025 Dec 10;16:1697712. doi: 10.3389/fimmu.2025.1697712 (PMC12728067; doi:10.3389/fimmu.2025.1697712)
Supplement: Supplementary file 1 [file Table1.docx]

| **Patient Number** | **Group** | **First-line treatment regimen** | **Second-line treatment regimen** |
| --- | --- | --- | --- |
| 001 | Immunotherapy Rechallenge | Nivolumab+XELOX | Nivolumab+Nab-PTX |
| 002 | Immunotherapy Rechallenge | Tislelizumab+SOX | Tislelizumab+Nab-PTX |
| 003 | Chemotherapy | Tislelizumab+PS | FOLFOX |
| 004 | Immunotherapy Rechallenge | Sintilimab+XELOX | Sintilimab+Nab-PCb |
| 005 | Immunotherapy Rechallenge | Sintilimab+SOX | Cadonilimab+Nab-PTX |
| 006 | Chemotherapy | Tislelizumab+XELOX | Nab-PCb |
| 007 | Immunotherapy Rechallenge | Camrelizumab+XELOX | Camrelizumab+Nab-PS |
| 008 | Immunotherapy Rechallenge | Tislelizumab+XELOX | Tislelizumab+Nab-PS |
| 009 | Immunotherapy Rechallenge | Sintilimab+SOX | Sintilimab+FOLFOX |
| 010 | Chemotherapy | Sintilimab+SOX | Docetaxel+Cisplatin |
| 011 | Chemotherapy | Tislelizumab+FOLFOX | Nab-PTX |
| 012 | Chemotherapy | Sintilimab+FOLFOX | SOX |
| 013 | Immunotherapy Rechallenge | Sintilimab+FOLFOX | Tislelizumab+FOLFOX |
| 014 | Chemotherapy | Sintilimab+SOX | Nab-PS |
| 015 | Chemotherapy | Sintilimab+SOX | Nab-PS |
| 016 | Chemotherapy | Sintilimab+SOX | Nab-PS |
| 017 | Immunotherapy Rechallenge | Sintilimab+SOX | Pembrolizumab+Irinotecan |
| 018 | Immunotherapy Rechallenge | Sintilimab+SOX | Tislelizumab+Nab-PCb |
| 019 | Chemotherapy | Tislelizumab+SOX | XELOX |
| 020 | Immunotherapy Rechallenge | Sintilimab+SOX | Sintilimab+Nab-PTX +P |
| 021 | Immunotherapy Rechallenge | Sintilimab+XELOX | Cadonilimab+Nab-PTX |
| 022 | Immunotherapy Rechallenge | Sintilimab+XELOX | Sintilimab+Nab-PTX+Capecitabine |
| 023 | Immunotherapy Rechallenge | Sintilimab+SOX | Sintilimab+Nab-PTX+Capecitabine |
| 024 | Immunotherapy Rechallenge | Sintilimab+XELOX | Tislelizumab+Nab-PTX |
| 025 | Chemotherapy | Sintilimab+XELOX | Nab-PCb |
| 026 | Immunotherapy Rechallenge | Nivolumab+XELOX | Nivolumab+Nab-PTX+ Capecitabine |
| 027 | Immunotherapy Rechallenge | Sintilimab+SOX | Sintilimab+Nab-PTX+ Capecitabine |
| 028 | Immunotherapy Rechallenge | Sintilimab+XELOX | Cadonilimab+Nab-PTX |
| 029 | Chemotherapy | Sintilimab+SOX | Nab-PTX+ Capecitabine |
| 030 | Immunotherapy Rechallenge | Sintilimab+XELOX | Sintilimab+Nab-PTX |
| 031 | Immunotherapy Rechallenge | Sintilimab+nab-XC | Cadonilimab+Irinotecan |
| 032 | Immunotherapy Rechallenge | Sintilimab+SOX | Tislelizumab+Nab-PTX+Capecitabine |
| 033 | Immunotherapy Rechallenge | Sintilimab+XELOX | Sintilimab+Nab-PTX |
| 034 | Immunotherapy Rechallenge | Sintilimab+XELOX | Cadonilimab+Nab-PTX |
| 035 | Chemotherapy | Camrelizumab+PS | FOLFOX |
| 036 | Immunotherapy Rechallenge | Sintilimab+XELOX | Pembrolizumab+Nab-PTX+ Capecitabine |
| 037 | Immunotherapy Rechallenge | Sintilimab+XELOX | Sintilimab+Nab-PTX |
| 038 | Immunotherapy Rechallenge | Sintilimab+SOX | Cadonilimab+IRI + Cap |
| 039 | Immunotherapy Rechallenge | Sintilimab+SOX | Sintilimab+FOLFOX |
| 040 | Immunotherapy Rechallenge | Nivolumab+SOX | Nivolumab+Docetaxel +Cisplatin |
| 041 | Immunotherapy Rechallenge | Sintilimab+FOLFOX | Sintilimab+S-1 |
| 042 | Immunotherapy Rechallenge | Sintilimab+SOX | Cadonilimab+Nab-PTX |
| 043 | Chemotherapy | Sintilimab+XELOX | Nab-PS |
| 044 | Immunotherapy Rechallenge | Nivolumab+SOX | Cadonilimab+Docetaxel+Cisplatin |
| 045 | Immunotherapy Rechallenge | Sintilimab+SOX | Sintilimab+Nab-PS |
| 046 | Chemotherapy | Sintilimab+nab-PC | Nab-PS |
| 047 | Immunotherapy Rechallenge | Tislelizumab+SOX | Tislelizumab+Nab-PS |
| 048 | Chemotherapy | Sintilimab+SOX | Nab-PTX |
| 049 | Chemotherapy | Sintilimab+SOX | Nab-PTX+Capecitabine |
| 050 | Chemotherapy | Sintilimab+SOX | Nab-PS |
| 051 | Immunotherapy Rechallenge | Sintilimab+XELOX | Sintilimab+SOX |
| 052 | Immunotherapy Rechallenge | Sintilimab+SOX | Sintilimab+SOX |
| 053 | Chemotherapy | Sintilimab+SOX | Nab-PTX |
| 054 | Chemotherapy | Sintilimab+nab-PS | Nab-PCb |
| 055 | Chemotherapy | Sintilimab+SOX | Nab-PCb |
| 056 | Chemotherapy | Sintilimab+SOX | Nab-PCb |
| 057 | Immunotherapy Rechallenge | Sintilimab+SOX | Sintilimab+Nab-PCb |
| 058 | Immunotherapy Rechallenge | Sintilimab+nab-PS | Sintilimab+Cisplatin+5-FU |
| 059 | Chemotherapy | Sintilimab+SOX | Nab-PCb |
| 060 | Immunotherapy Rechallenge | Sintilimab+XELOX | Sintilimab+Nab-PTX |
| 061 | Immunotherapy Rechallenge | Sintilimab+SOX | Sintilimab+Nab-PTX |
| 062 | Immunotherapy Rechallenge | Sintilimab+SOX | Sintilimab+Nab-PCb |
| 063 | Chemotherapy | Sintilimab+SOX | Nab-paclitaxel+Capecitabine |
| 064 | Chemotherapy | Sintilimab+SOX | Nab-paclitaxel+Capecitabine |
| 065 | Immunotherapy Rechallenge | Sintilimab+XELOX | Sintilimab+Nab-PS |
| 066 | Chemotherapy | Sintilimab+XELOX | Nab-PCb |
| 067 | Immunotherapy Rechallenge | Sintilimab+nab-PS | Sintilimab+Nab-PCb |
| 068 | Immunotherapy Rechallenge | Sintilimab+XELOX | Sintilimab+Nab-PCb |
| 069 | Immunotherapy Rechallenge | Sintilimab+SOX | Sintilimab+Nab-PCb |
| 070 | Chemotherapy | Sintilimab+SOX | Nab-paclitaxel+Ccapecitabine |
| 071 | Chemotherapy | Sintilimab+XELOX | Nab-PS |
| 072 | Immunotherapy Rechallenge | Sintilimab+SOX | Sintilimab+Nab-PCb |
| 073 | Chemotherapy | Sintilimab+nab-PC | XELOX |
| 074 | Chemotherapy | Sintilimab+XELOX | Nab-PCb |
| 075 | Chemotherapy | Sintilimab+SOX | Nab-PCb |
| 076 | Chemotherapy | Sintilimab+SOX | Docetaxel+Cisplatin |
| 077 | Chemotherapy | Sintilimab+nab-PC | Docetaxel |
| 078 | Immunotherapy Rechallenge | Sintilimab+SOX | Sintilimab+Nab-PCb |
| 079 | Immunotherapy Rechallenge | Sintilimab+nab-PC | Sintilimab+Irinotecan |
| 080 | Chemotherapy | Sintilimab+XELOX | Docetaxel+Cisplatin |
| 081 | Chemotherapy | Sintilimab+XELOX | Nab-PCb |
| 082 | Immunotherapy Rechallenge | Sintilimab+XELOX | Sintilimab+Nab-paclitaxel+Capecitabine |
| 083 | Immunotherapy Rechallenge | Sintilimab+XELOX | Sintilimab+Nab-PS |
